# 

**Published:** 1918-02

**Authors:** 


### TABLE OF CONTENTS

The Imperfect Sight of the Normal Eye ---------------- 317

The Differentiation of Abdominal Pain and Its Bearing on Treatment ---------------------------------- 323

Defective Nutrition in Early Life ------------------- 333

Supurative Appedicitis -------------------------------- 343

Medical Items ------------------------------------------ 349

